# COVID-19 in Brazil: advantages of a socialized unified health system and preparation to contain cases

**DOI:** 10.1590/0037-8682-0167-2020

**Published:** 2020-04-17

**Authors:** Julio Croda, Wanderson Kleber de Oliveira, Rodrigo Lins Frutuoso, Luiz Henrique Mandetta, Djane Clarys Baia-da-Silva, José Diego Brito-Sousa, Wuelton Marcelo Monteiro, Marcus Vinícius Guimarães Lacerda

**Affiliations:** 1 Universidade Federal do Mato Grosso do Sul, Faculdade de Medicina, Campo Grande, MS, Brasil.; 2 Fundação Oswaldo Cruz, Campo Grande, MS, Brasil.; 3 Ministério da Saúde, Secretária de Vigilância em Saúde, Departamento de Imunizações e Doenças Transmissíveis, Brasília, DF, Brasil.; 4 Ministério da Saúde, Secretária de Vigilância em Saúde, Brasília, DF, Brasil.; 5 Ministério da Saúde, Departamento de Saúde Ambiental e Saúde do Trabalhador, Brasília, DF, Brasil.; 6 Ministério da Saúde, Brasília, DF, Brasil; 7 Universidade do Estado do Amazonas, Manaus, AM, Brasil.; 8 Fundação de Medicina Tropical Doutor Heitor Vieira Dourado, Manaus, AM, Brasil.; 9 Fundação Oswaldo Cruz, Instituto Leônidas & Maria Deane, Manaus, AM, Brasil.

**Keywords:** COVID19, Brazil, Socialized unified health system, Measures

## Abstract

The outbreak of new coronavirus disease 2019 (COVID-19) reported for the first time in Wuhan, China in late December 2019 have rapidly spread to other countries and it was declared on January 30, 2020 as a public health emergency of international concern (PHEIC) by the World Health Organization. Before the first COVID-19 cases were reported in Brazil, several measures have been implemented including the adjustment of legal framework to carry out isolation and quarantine. As the cases increased significantly, new measures, mainly to reduce mortality and severe cases, have also been implemented. Rapid and robust preparedness actions have been undertaken in Brazil while first cases have not yet been identified in Latin-American. The outcome of this early preparation should be analyzed in future studies.

## INTERNATIONAL PUBLIC HEALTH PROBLEMS AND EMERGENCY HEALTH OPERATIONS CENTERS

In the last two decades, the world has undergone important changes that impact health and the economy at individual and global levels, and these reflect directly on the public health of populations of many countries ^1^ . The recently emerged SARS-CoV-2 pandemic, with first cases reported in Wuhan, China in late December, 2019, quickly spread to other countries ^2^ and was declared by the World Health Organization (WHO) as of January 30, 2020, an Public Health Emergency of International Concern (PHEIC) ^3^ ^,^ ^4^ . PHEIC are extraordinary events which pose a large scale public health risk with international spreading and which, in general, require a coordinated response ^5^ . In Brazil, national public health emergencies (NPHE) are defined according to Brazilian Ministry of Health (MoH) as events that represent risks to public health and that occur in situations of outbreaks or epidemics (as a result of unexpected agents or reintroduction of eradicated diseases or with high severity), disasters and of lack of assistance to the population, which go beyond the response capacity of the state ^6^ .

In our current pandemic scenario, which represents an important NPHE, the promotion of actions and quick responses is necessary and Emergency Health Operations Centers (EHOCs) play an important role. When necessary, EHOCs are activated and work continuously in an organizational structure by monitoring and analyzing epidemiological data and field reports from various sources in order to support the decision making of managers and technicians in the definition of appropriate and timely strategies and actions for coping with such public health emergencies ^7^ ^,^ ^8^ . In Brazil, the health surveillance secretariat (HSS) is responsible for activating EHOCs, based on the Event Monitoring Committee’s (EMC) recommendations, as well as for classifying the emergency level (zero, I, II, III) ^9^ .

## HOW DID BRAZIL RESPOND TO COVID-19?

In the context of COVID-19 in China and the provisions of decree MH No. 2,952 of December 14, 2011 ^10^ , NPHE was declared in Brazil on January 10, 2020. On January 22 the Brazil’s MoH, via Decree No.188 ^11^ activated the EHOC-nCoV operations center, with alert level 1 (no suspected cases at the time), which was coordinated by HSS. The fundamental objective of EHOC-nCoV was to respond to the SARS-CoV-2 emergency at the national level by organizing a coordinated action within the scope of UHS. In addition, EHOC-nCoV would advise states and municipalities secretaries of health and the federal government, public and private health services, agencies and companies regarding contingency plans and response measures that should be proportional and restricted to the current risks ^12^ . On January 27 the first suspected coronavirus case in Brazil was identified, leading to raising the alert level to level 2 (imminent risk).

On January 28, the first EHOC-nCoV Epidemiologic Bulletin ^13^ , epidemiological surveillance guideline and National Contingency Plan (NCP) for the COVID-19 with alert levels were published ^13^ . Epidemiological surveillance aims at guiding the National Health Surveillance System and the UHS service network to act in the identification of COVID-19 cases in order to mitigate the risks of sustained transmission and the appearance of severe cases and subsequent deaths ^12^ . The epidemiological surveillance and NCP are based on structured documents and evidence accumulated by other countries including China, in epidemics such as SARS-CoV, MERS-CoV and SARS-CoV-2, which had never occurred before in Brazil. However, Brazil had previous experience with other respiratory virus pandemics, such as H1N1, which started in 2009 and was responsible for 46,355 cases registered in the country until March 2010. In addition, NCP actions are based on national and state plans for surveillance and clinical management of severe acute respiratory syndrome (SARS) and flu syndrome (FS) ^13^ .

All states in the country were encouraged to adapt the NCP based on their infrastructure and regional characteristics, as well as to provide for actions to combat the disease in their territories. It is important to highlight that the NCP is based on the information made available by WHO (based on compilation of information received by different countries) and on scientific evidence, and therefore the NCP procedures undergo necessary changes ^14^ . Risks should be assessed and reviewed periodically, with a view to developing scientific knowledge and adoption of locally appropriate measures ^13^ .

On January 30, COVID-19 was declared a Public Health Emergency of International Concern (PHEIC) by WHO. The Brazilian Interministerial Executive Group on Public Health (IEG-PHE) was reactivated through Decree No. 10,211 (January 30). Its main attributions are i) to propose, monitor and articulate preparedness and coping measures, allocation of budgetary-financial resources to implement the necessary measures; ii) to establish guidelines for the definition of local criteria for monitoring the implementation of emergency measures and iii) to prepare reports on the public health emergency situation and diseminate to ministers ^15^ .

Brazil declared COVID-19 a public health emergency (PHE) on February 3, and on February 6 the MoH approved the law No.13,979 ^16^ (Quarantine Law), with measures aimed at protecting the community and dealing with PHE resulting from SARS-CoV-2, including isolation; quarantine; compulsory notification, epidemiological study or investigation; exhumation, necropsy, cremation and corpse management; exceptional and temporary restriction on entering and leaving the country; requisition of goods and services from natural and legal persons, in which case the subsequent payment of fair compensation will be guaranteed. However, these measures can only be determined based on scientific evidence and analysis of strategic health information.

The first case of coronavirus in Brazil and in South America ^17^ was registered on February 26, 2020 in São Paulo. It was a 61-year-old man with a history of travel to the Lombardy region, Italy, which had reported a high number of cases and deaths. The number of cases has increased since in the territory, and several measures have now been taken. On March 13, MoH and professionals from the state health departments across the country announced recommendations to prevent the spread of the disease, as previously determined in Decree No. 356 of March 11 ^18^ ^,^ ^19^ . MoH recognized that community transmission was occurring across the country on March 20, as a strategic measure to ensure a collective effort by all Brazilians in order to reduce the virus transmission ^20^ . Implementation of nom-pharmacological measures, including physical distancing and quarantine required the determination of community transmission countrywide by the MoH. Quarantine has been controversial and must be evaluated very carefully, taking into account the COVID-19 epidemic progression in China, Italy and Spain.

Currently, the disease has shown an increase in the number of cases, and as of March 31, 5,933 reported cases and 206 deaths had been registered in Brazil. São Paulo has been the most affected state, with 136 deaths and 2,339 confirmed cases, followed by Rio de Janeiro with 23 deaths and 708 confirmed cases. On March 27, MoH made official (Note nº 5/2020-DAF/SCTIE/MS ^21^ ) the use of chloroquine (CQ) and hydroxychloroquine (HCQ) in patients with severe forms of COVID-19 ^22^ ^-^ ^27^ . The proposed protocol consists of treatment over five days, however these two drugs should be used as a complementary measure to all other types of treatment support used, such as mechanical ventilation and symptomatic medications, as well as others provided in the treatment manual ^21^ . Two national clinical studies to evaluate the effectiveness of the CQ use as treatment for COVID-19 infection were approved by the national research ethics committee (CONEP) ^28^ .

## PROGRESSION OF CASES IN BRAZIL AND MISTAKES ALONG THE WAY

**FIGURE 1:**
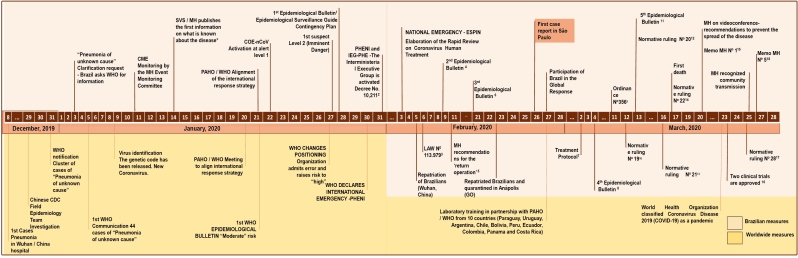
Evolution of the Coronavirus emergency and response from the Braziliam Ministry of Health (Adapatated from: https://www.saude.gov.br/images/pdf/2020/fevereiro/04/Boletim-epidemiologico-SVS-04fev20.pdf ) *1st Epidemiological Bulletin: International monitoring event in Chin. ( https://portalarquivos2.saude.gov.br/images/pdf/2020/janeiro/15/Boletim-epidemiologico-SVS-01.pdf ) 1. http://www.planalto.gov.br/ccivil_03/_ato2019-2022/2020/lei/L13979.htm 2. http://www.planalto.gov.br/ccivil_03/_ato2019-2022/2020/decreto/D10211.htm 3. Relates to measures for dealing with the public health emergency of international importance resulting from the coronavirus responsible for the 2019 outbreak ( http://www.planalto.gov.br/ccivil_03/_ato2019-2022/2020/lei/L13979 ). 4. https://portalarquivos2.saude.gov.br/images/pdf/2020/fevereiro/13/Boletim-epidemiologico-COEcorona-SVS-13fev20.pdf 5. https://portalarquivos2.saude.gov.br/images/pdf/2020/fevereiro/11/operacao-regresso-11fev-b.pdf 6. https://www.saude.gov.br/images/pdf/2020/fevereiro/21/2020-02-21-Boletim-Epidemiologico03.pdf 7. Treatment Protocol ( https://www.arca.fiocruz.br/bitstream/icict/40195/2/Protocolo_Tratamento_Covid19.pdf ) 8. https://www.saude.gov.br/images/pdf/2020/marco/04/2020-03-02-Boletim-Epidemiol--gico-04-corrigido.pdf 9. Relates to the regulation and operationalization of the provisions of Law No. 13,979, of February 6th, 2020, which establishes the measures to overcome the public health emergency of international importance resulting from the coronavirus (COVID-19) ( http://www.in.gov.br/en/web/dou/-/portaria-n-356-de-11-de-marco-de-2020-247538346 ) 10. Establishes guidelines for the bodies and entities of the Civil Personnel System of the Federal Public Administration - SIPEC, regarding the protection measures for overcoming the public health emergency of international importance resulting from the coronavirus (COVID-19). http://www.in.gov.br/en/web/dou/-/instrucao-normativa-n-19-de-12-de-marco-de-2020-247802008 ) 11. https://www.saude.gov.br/images/pdf/2020/marco/24/03--ERRATA---Boletim-Epidemiologico-05.pdf 12. Amends the Normative Ruling No. 19 ( http://www.in.gov.br/en/web/dou/-/instrucao-normativa-n-20-de-13-de-marco-de-2020-247887393 ) 13. Amends the Normative Ruling No. 19 ( http://www.in.gov.br/en/web/dou/-/instrucao-normativa-n-21-de-16-de-marco-de-2020-248328867 ) 14. Establishes guidelines for the bodies and entities of the Civil Personnel System of the Federal Public Administration - SIPEC, regarding the protection measures to overcome the public health emergency of international importance resulting from COVID-19, related to the process of re-registering retirees, pensioners and civilian politicians ( http://www.in.gov.br/en/web/dou/-/instrucao-normativa-n-22-de-17-de-marco-de-2020-248564245 ) 15. Memo MH Nº 114- Coronavirus and risk in patients with Hereditary Hemorrhagic Diseases ( https://www.saude.gov.br/images/pdf/2020/marco/19/SEI-MS---0014038615---Nota-Informativa.pdf ) 16. Two clinical trials are approved to assess the effectiveness of chloroquine in critically ill patients ( https://conselho.saude.gov.br/images/BOLETIM_EP_EdEspecialCoronavirus_23marco2020.pdf ) 17. Establishes guidelines for the bodies and entities of the Civil Personnel System of the Federal Public Administration - SIPEC, regarding the authorization for extraordinary service, the granting of transport assistance, night allowances and occupational allowances to public servants and employees who perform their activities remotely or who are away from their face-to-face activities, under the terms of Normative Ruling No. 19, of March 12th, 2020, and to take other measures ( http://www.in.gov.br/en/web/dou/-/instrucao-normativa-n-28-de-25-de-marco-de-2020-249807751 ) 18. Chloroquine as an adjunct therapy in the treatment of critically-ill patients. ( https://12ad4c92-89c7-4218-9e11-0ee136fa4b92.filesusr.com/ugd/3293a8_49de9bf961b846708f91cb03dfe076bc.pdf )

The number of cases in Brazil is growing rapidly. Several measures had been taken by MoH even before the first case was registered in the country, as previously described and shown in Figure 1 . It is important to note, however, that on January 27 WHO admitted a significant error associated with COVID-19 global risk assessment, which until three days earlier was considered moderate, however the disease was considered of very high risk in China, while at high regional and global levels. This may have hindered measures to implement specific international interventions in a timely manner and may have resulted in an increase in the number of cases in China and the spread of the disease to other countries, including Brazil.

In this sense, Brazil has been following WHO recommendations and recent scientific evidence generated by China and Italy ^29^ . However, it is important to note that Brazil has distinct and peculiar characteristics, including population structure. It is a country whose population consists mainly of young adults. In addition, comorbidities and co-infections, such as diabetes, hypertension, HIV, tuberculosis, obesity, among others, are prevalent. Thus, it is potentially important that the younger population with comorbidities/co-infections are not neglected.

In addition, it is important to note that fall is coming in the next days in the southern half of Brazil. During fall and winter seasons, the incidence of respiratory diseases increase (cold, flu, asthma attacks, sinusitis, pneumonia, bronchitis) and currently COVID-19 should be added to this list. Drier air and lower temperature may lead to an increase in the risk of coronavirus transmission and number of COVID-19 cases. Because symptoms of flu and SARS-CoV-2 are similar, MoH anticipated the usual free vaccination for influenza, for major risk groups, in order to help health professionals rule out influenza in patient screening and improve diagnosis of the new virus.

## BRAZIL’S EXPERIENCE WITH OTHER HEALTH EMERGENCIES

Brazil has already experienced other public health emergencies with diseases including polio, smallpox, cholera, H1N1 (influenza A), avian influenza, yellow fever, severe acute respiratory syndrome, and zika. Many of these emergencies marked the history of Brazilian public health policy and led to the implementation of control and eradication measures, such as for smallpox, which was eradicated in 1977. Among the most recent public health emergency diseases, the H1N1 epidemic in 2009 and the Zika epidemic in 2015-2016 are noteworthy. Both constitute an important legacy of how to deal with epidemics; the latter (Zika) demonstrated Brazil’s scientific leadership due to the association of infection with cases of microcephaly ^30^ .

The H1N1 pandemic helped UHS improve its capacity to respond to emergencies due to respiratory syndromes (RS), an ongoing process since 2005. Currently, UHS has plans, protocols, procedures and guides for identifying, monitoring and responding to emergencies due to RS. Many recommended procedures, mainly those included in the influenza chapter of the Epidemiological Surveillance Guide, are applied in the context of suspected cases of Coronavirus ^12^ ^,^ ^14^ . However, the initial recommendation is to discard the most common respiratory diseases and adopt the flu treatment protocol in a timely manner to avoid serious cases and deaths from known respiratory diseases, when indicated. The UHS has the capacity and experience to respond to RS-related emergencies and currently, with the new Coronavirus protocol, it has been possible to adjust some recommendations to the specific context of the COVID-19 emergency. These adjustments are based on the information made available by WHO on a daily basis and every procedure is susceptible to the necessary changes and its adequacy may be fundamental to deal with the next pandemics that are likely to occur ^14^ .

In addition to that, the country counts on a decentralized network of central laboratories in each state (LACENs), with existing capacity, and a public manufacture chain of laboratory supplies for diagnostic RT-PCR, e.g., in Fiocruz (Biomanguinhos). In case of evidence of CQ efficacy, Farmanguinhos and LQFEx are already public producers of CQ diphosphate for malaria treatment.

During the Zika epidemic, Brazil led the discovery and reported the relationship between the Zika virus and the increase in cases of microcephaly. The first reports of increased cases of microcephaly occurred in the state of Pernambuco, in October, 2015 ^30^ ^,^ ^32^ . As soon as it was discovered by the state health departments, the MoH sent technical teams to help with the investigations and notified WHO of the situation ^30^ . Once the association between Zika and microcephaly in Brazil, where it occurred first, was confirmed, the first version of the plan to fight *Aedes* spp. and microcephaly was published in December 2015 ^31^ ^,^ ^32^ . WHO recognized Brazil’s main role in this critical finding. In May 2017, a risk assessment concluded that Brazil no longer met the criteria for defining an emergency, according to WHO parameters.

## COVID-19: THE WAY FORWARD

Although Brazil is attempting to implement measures to reduce the number of cases, mainly focused on physical distancing, an increase in COVID-19 cases is expected in the coming months. Several mathematical models have shown that the virus will be potentially circulating until mid-September, with an important peak of cases in April and May. Thus, there are concerns regarding availability of intensive care units (ICUs) and mechanical ventilators necessary for patients hospitalized with COVID-19 as well as the availability of specific diagnostic tests, particularly real time RT-PCR, for the early detection of COVID -19 and the prevention of subsequent transmission. RT-PCR increased capacity and serologic/RDT tests may become available soon, in part due to the private and public/academia collaboration/contribution (e.g., Farmanguinhos, Vale). Virus sequencing has been performed by sentinel sites and molecular biologists interact intensely now.

Regarding cultural differences, the use of masks is common and accepted in Asia, none existing in Latin America. This means both culturally accepted and daily routine, but also people can buy them easily there, as well as bowing more there, much more physical contact in our cultures. These differences might be decisive in the evolution of the pandemics, and also need to be addressed in social sciences protocols.

Physical distancing is a measure that should be suggested early in order to flatten the epidemiological curve with the least possible economic impact. By the end of March 2020, Brazilian authorities still maintain the recommendation of physical distancing and have not implemented a lockdown through the use of security forces to prevent mass movement of people. If physical distancing is effective by limiting the public’s access to essential services only, the economic impact can be mitigated while the current COVID-19 epidemic is controlled.
